# Supplementary material for: Exciton fission in monolayer transition metal dichalcogenide semiconductors
Source: Nat Commun. 2017 Oct 27;8:1166. doi: 10.1038/s41467-017-01298-6 (PMC5660116; doi:10.1038/s41467-017-01298-6)
Supplement: Supplementary file 1 — Supplementary Information [file 41467_2017_1298_MOESM1_ESM.pdf]

Supplementary Note 1

Ab-initio based parametrization of the transition metal dichalcogenides

To get an *analytic description* of the Coulomb interaction in semiconducting transition metal dichalcogenide (TMDC) monolayers, we apply the approach as presented in [1–3] for MoS_2 also to $MoSe_2$, WS_2, and WSe_2. We start with ab initio calculations for density-density like bare $U_{\alpha\beta}(q)$ and screened $V_{\alpha\beta}(q)$ Coulomb interaction matrix elements in the Wannier basis (with $\alpha, \beta \in [d_{z^2}, d_{xy}, d_{x^2-y^2}]$) for the freestanding, undoped TMDC slabs using the FLEUR and SPEX codes [4–6] on discrete $18 \times 18 \times 1$ q grids. To interpolate the resulting 3×3 matrices analytically we make use of the (sorted) eigenbasis of the bare Coulomb interaction $\mathbf{U}$ by diagonalizing it

$$\mathbf{U}_{\text{diag}}(q) = \begin{pmatrix} U_1(q) & 0 & 0 \\ 0 & U_2 & 0 \\ 0 & 0 & U_3 \end{pmatrix}, \tag{1}$$

where the diagonal matrix elements are given by

$$U_i = \langle e_i | \mathbf{U} | e_i \rangle \tag{2}$$

using the eigenvectors of $\mathbf{U}$ in their long-wavelength limits

$$e_1 = \frac{1}{\sqrt{3}} \begin{pmatrix} 1 \\ 1 \\ 1 \end{pmatrix}, e_2 = \frac{1}{\sqrt{6}} \begin{pmatrix} +2 \\ -1 \\ -1 \end{pmatrix}, e_3 = \frac{1}{\sqrt{2}} \begin{pmatrix} 0 \\ +1 \\ -1 \end{pmatrix}. \tag{3}$$

While the leading eigenvalue $U_1(q)$ is a function of q the other two eigenvalues can be readily approximated as constants (see Fig. 1). For the analytic description of the former we use

$$U_1(q) = \frac{3e^2}{2\varepsilon_0 A} \frac{1}{q(1 + \gamma q + \delta q^2)}, \tag{4}$$

where $A = \frac{\sqrt{3}}{2} a^2$ is the area of the hexagonal unit cell, a is the lattice constant, and ε_0 is the vacuum permittivity.

The matrix elements of the screened interaction $\mathbf{V}(q)$ in the eigenbasis of the bare interaction $\mathbf{U}(q)$ are then obtained via

$$V_i(q) = \varepsilon_i^{-1}(q)\, U_i(q) \tag{5}$$

where $\varepsilon_i(q)$ accounts for both the material-specific internal polarizability and the screening by the environment. Its diagonal representation is given by

$$\varepsilon_{\text{diag}}(q) = \begin{pmatrix} \varepsilon_1(q) & 0 & 0 \\ 0 & \varepsilon_2 & 0 \\ 0 & 0 & \varepsilon_3 \end{pmatrix}. \tag{6}$$

Once again, the leading eigenvalue $\varepsilon_1(q)$ is a function of q while the other elements are described sufficiently well as constants. Here, the former can be expressed by

$$\varepsilon_1(q) = \varepsilon_\infty(q) \frac{1 - \beta_1 \beta_2 e^{-2q\,h}}{1 + (\beta_1 + \beta_2) e^{-q\,h} + \beta_1 \beta_2 e^{-2q\,h}} \tag{7}$$

which describes the macroscopic dielectric function of a two-dimensional semiconductor. The parameters β_i include the screening effects of substrates (see Ref. [7]) via

$$\beta_i = \frac{\varepsilon_\infty(q) - \varepsilon_{\text{sub},i}}{\varepsilon_\infty(q) + \varepsilon_{\text{sub},i}} \tag{8}$$

where the dielectric constants of the substrate ($i = 1$) and the superstrate ($i = 2$) are introduced. In order to describe the original ab initio data as close as possible we fit $\varepsilon_\infty(q)$ using

$$\varepsilon_\infty(q) = \frac{a + q^2}{\frac{a \sin(qc)}{qbc} + q^2} + e \tag{9}$$

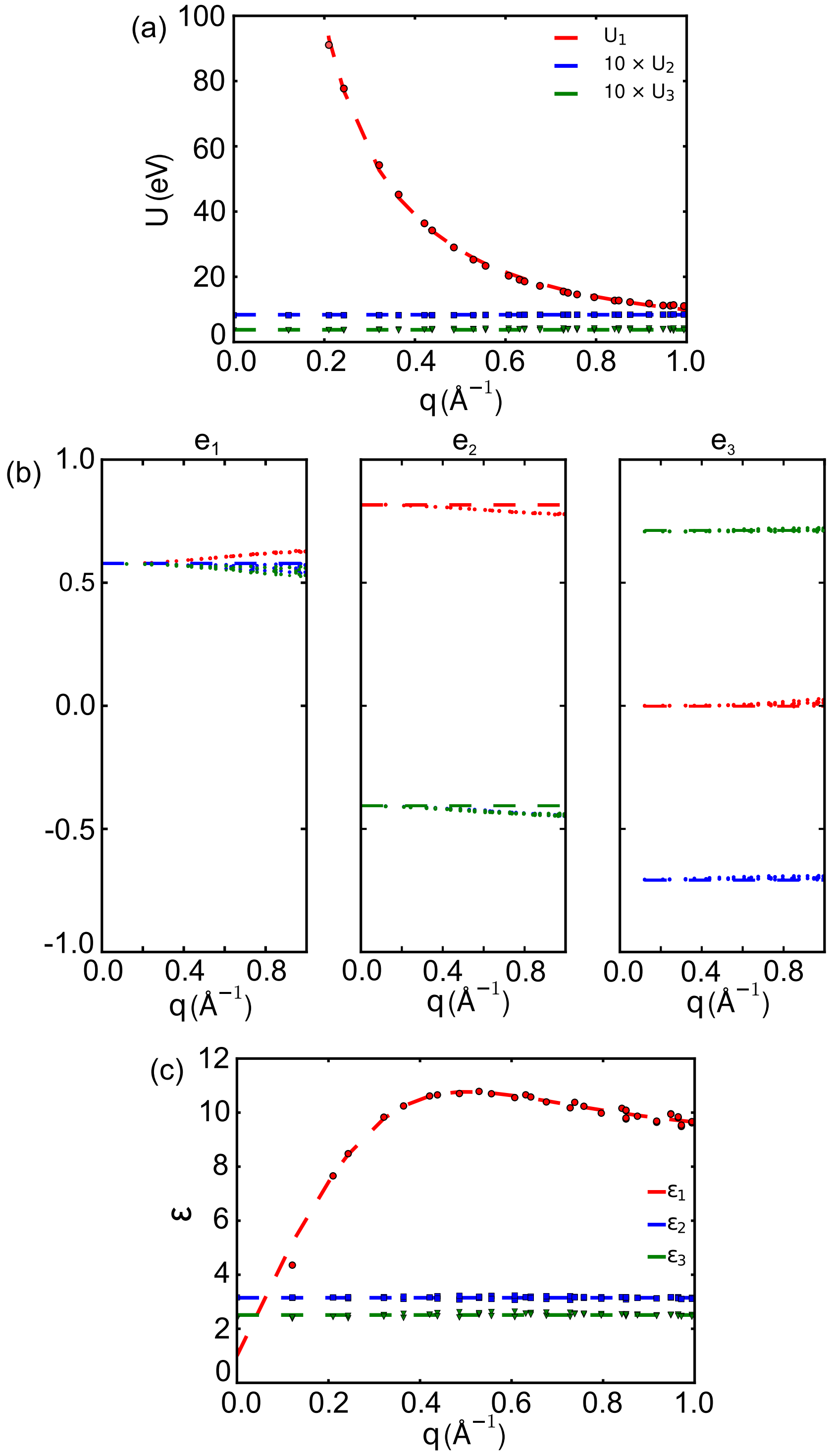

Supplementary Figure 1: **Examplary data for the fit and ab initio calculation of the Coulomb interaction in $MoSe_2$.** **(a)** Bare Coulomb matrix elements in its eigenbasis. Red dots, blue squares and green triangles correspond to the leading, second and third eigenvalue of $U(q)$ as obtained from ab initio calculations. Dashed lines show the corresponding fits using Supplementary Equation (4) and Supplementary Table I. **(b)** Eigenvectors of the bare Coulomb matrix (from left to right corresponding to the leading, second and third eigenvalue). The corresponding vector elements of the d_{z^2} (red), d_{xy} (green) and $d_{x^2-y^2}$ (blue) orbitals are shown. Dashed lines indicate constant analytic values for $U(q \to 0)$, see Supplementary Equation (3). **(c)** Matrix elements of the diagonal dielectric function. Markers indicate ab initio results and dashed lines show the fits using Supplementary Equation 7 and Supplementary Table I.

Supplementary Table I: **Parameters describing the Coulomb interaction and the static screening in the semiconducting TMDCs MX_2.**

	MoS_2	$MoSe_2$	WS_2	WSe_2
lattice constant a(Å)	3.180	3.320	3.191	3.325
bare Interaction U				
γ (Å)	1.932	2.232	2.130	2.297
δ (Å^2)	0.395	-0.356	0.720	0.174
A (Å^2)	8.758	9.546	8.818	9.574
U_2 (eV)	0.810	0.837	0.712	0.715
U_3 (eV)	0.367	0.376	0.354	0.360
screening ε				
a (1/Å^2)	2.383	2.856	3.947	2.430
b	17.836	11.635	29.931	20.764
c (Å)	5.107	1.979	5.440	5.761
h (Å)	2.740	4.298	1.578	2.489
e	5.739	6.303	4.497	5.305
ε_2	3.077	3.148	2.979	3.028
ε_3	2.509	2.510	2.494	2.481

and $\varepsilon_{\text{sub},1} = \varepsilon_{\text{sub},2} = 1$ as the ab intio calculations were performed for freestanding layers.

As soon as all fitting parameters are obtained (see Supplementary Table I) the screening of a dielectric environment can be included by choosing $\varepsilon_{\text{sub},1}$ or $\varepsilon_{\text{sub},2}$ correspondingly. Thus we have a closed analytic description of the screened Coulomb interaction $\mathbf{V}(q)$ in the eigenbasis of the bare interaction $\mathbf{U}(q)$ at arbitrary momenta q in the first Brillouin zone. In order to transform it to the original orbital basis we make use of the eigensystem given in Supplementary Equation (3). In fact, this model is appropriate for every two-dimensional semiconductor.

Besides the analytical model of the screened Coulomb matrix elements we make use of a tight-binding Hamiltonian to describe the electronic band structure (as obtained from G_0W_0 calculations) of the TMDC slab. To this end, we utilize the same Wannier basis as in Ref. 8 and derive a minimal three-band model containing the highest valence and two lowest conduction bands using the Wannier90 package [9]. Thereby we solely disentangle our target bands from the rest without performing a maximal localization in order to preserve the original transition metal d-orbital characters. The latter is crucial for the subsequent addition of first and second order Rashba spin-orbit coupling following Ref. [2], which takes into account the large spin-orbit splitting in the conduction- and the valence-band K valleys.

We use relaxed lattice constants as given in Supplementary Table I. The values of the bare and screened Coulomb interaction were extrapolated from vacuum heights of 16 Å to 32 Å.

Supplementary Note 2

Ab initio Two-Particle Properties

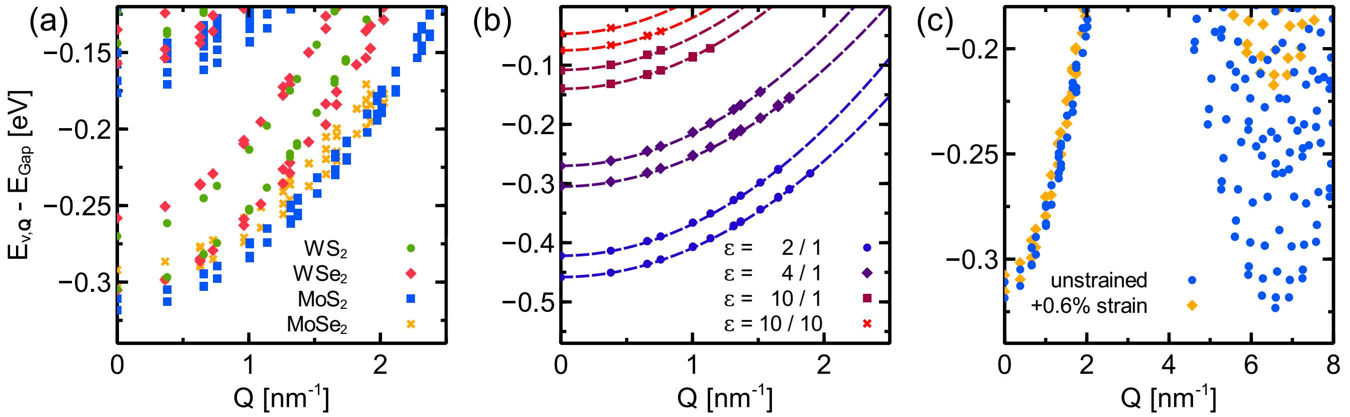

Supplementary Figure 2: **Bound-state spectra at zero excitation density.** The spectra are measured relative to the quasi-particle band gap and shown over the modulus of total exciton momentum Q. **(a)** Comparison of different TMDC materials on SiO_2 substrate. **(b)** Comparison of WS_2 in different dielectric environments with dielectric constant ε of bottom/top environment. Only 1s-excitons are shown. The lines serve as guide to the eye. **(c)** Comparison of MoS_2 with different lattice constants a on SiO_2 substrate. The unstrained layer corresponds to $a = 3.18$ Å while tensile biaxial strain is simulated by $a = 3.20$ Å .

For a material-realistic description of the ionization equilibrium in atomically thin systems, we use band structures and Coulomb matrix elements from material-realistic G_0W_0 calculations for freestanding slabs of various monolayer TMDCs as a basis, see the previous section. The matrix elements already take into account dielectric screening due to charge carriers in the ground state of the system. Furthermore, we account for additional screening provided by a possible dielectric environment, like a substrate, as described above. The main effect of the additional screening is a reduction of exciton binding energies, which are key quantities for the ionization equilibrium. The corresponding reduction of quasi-particle band gaps on the other hand plays no role here, as all bound-state energies and excited-carrier chemical potentials are measured relative to the gap, see the discussion and Fig. 4 in the main text. By convention, we choose the gap between the spin-up conduction and valence bands at K as reference which belong to the A-exciton resonance.

The calculation of ionization equilibrium requires knowledge of two-particle energies, which are obtained by solving the Bethe-Salpeter equation, Eq. (16) in the main text. As can be seen in Fig. 2 (a) the binding energies of the lowest $Q = 0$-excitons ("1s") are comparable for all considered materials, the main difference being the spin-orbit splitting between excitons involving different conduction bands, which is larger for tungsten-based materials. For MoS_2 bound states belonging to the B exciton are visible at -175 meV corresponding to the valence-band spin-orbit splitting of about 140 meV. Higher exciton states ("2s","2p") appear as well. Fig. 2 (b) shows that dielectric screening from the environment has a strong impact on the exciton spectrum as it can reduce exciton binding energies significantly [10]. This has a major influence on the ionization equilibrium, as we discuss in the main text. Another tunable parameter in experiments is the application of strain to the TMDC monolayer, which leads to changes in the band structure [11]. This is reflected by MoS_2 changing from an indirect to a direct semiconductor in the exciton picture under tensile strain, see Fig. 2 (c).

exciton dispersions shown in Fig. 2 and evaluate the sum over exciton states in polar coordinates. The lowest bound-state energies involving electrons and holes with equal spins are thus given by $E_{\mathbf{Q}} \approx E_{1s} + \hbar^2 Q^2/2M$, where we find $E_{1s} = -311$ meV and $M = 1.07 m_e$ for MoS_2 and $E_{1s} = -258$ meV and $M = 0.72 m_e$ for WSe_2. The energies E_{1s} are measured with respect to the quasi-particle band gap, while energy and momentum conservation in the exciton-photon interaction explicitely involve the band gap, restricting excitons to inside the light cone with radius $Q_{\max}$: $\hbar c Q_{\max} = E_{1s} + E_{\text{Gap}}$. The energy values on the right hand side correspond to the position of the A exciton in optical spectra, which we take from experiment [**?**] yielding 1.95 eV and 1.75 eV for MoS_2 and WSe_2 on SiO_2, respectively. The bright-exciton density is given by valleys. The smaller fraction of bright excitons in WSe_2 is mainly due to the fact that excitons with different electron and hole spins are energetically much lower due to the large conduction-band spin-orbit splitting.

Supplementary References

[1] Steinhoff, A., Rösner, M., Jahnke, F., Wehling, T. O. & Gies, C. Influence of excited carriers on the optical and electronic properties of MoS2. *Nano Lett.* **14**, 3743–3748 (2014).

[2] Liu, G.-B., Shan, W.-Y., Yao, Y., Yao, W. & Xiao, D. Three-band tight-binding model for monolayers of group-vib transition metal dichalcogenides. *Phys. Rev. B* **88**, 085433–085442 (2013).

[3] Schönhoff, G., Rösner, M., Groenewald, R. E., Haas, S. & Wehling, T. O. Interplay of screening and superconductivity in low-dimensional materials. *Phys. Rev. B* **94**, 134504–134510 (2016).

[4] The juelich fleur project. `http://www.flapw.de` (2014).

[5] Friedrich, C., Schindlmayr, A. & Blügel, S. Efficient calculation of the Coulomb matrix and its expansion around within the FLAPW method. *Comp. Phys. Comm.* **180**, 347–359 (2009).

[6] Friedrich, C., Blügel, S. & Schindlmayr, A. Efficient implementation of the gw approximation within the all-electron FLAPW method. *Phys. Rev. B* **81**, 125102–12517 (2010).

[7] Rösner, M., Şaşıoğlu, E., Friedrich, C., Blügel, S. & Wehling, T. O. Wannier function approach to realistic Coulomb interactions in layered materials and heterostructures. *Phys. Rev. B* **92**, 085102–085111 (2015).

[8] Groenewald, R. E., Rösner, M., Schönhoff, G., Haas, S. & Wehling, T. O. Valley plasmonics in transition metal dichalcogenides. *Phys. Rev. B* **93**, 205145–205151 (2016).

[9] Mostofi, A. A. *et al.* An updated version of wannier90: A tool for obtaining maximally-localised Wannier functions. *Comp. Phys. Comm.* **185**, 2309–2310 (2014).

[10] Latini, S., Olsen, T. & Thygesen, K. S. Excitons in van der waals heterostructures: The important role of dielectric screening. *Physical Review B* **92**, 245123–245135 (2015).

[11] Conley, H. J. *et al.* Bandgap engineering of strained monolayer and bilayer MoS2. *Nano Letters* **13**, 3626–3630 (2013).
